# The professionalism of ChatGPT in the field of surgery: low or high level?

**DOI:** 10.1097/JS9.0000000000001618

**Published:** 2024-05-09

**Authors:** Haiyang Wu, Wanqing Li, Xiaofeng Chen, Cheng Li

**Affiliations:** a Department of Orthopaedics, The First Affiliated Hospital of Zhengzhou University, Zhengzhou; b Department of Clinical College of Neurology, Neurosurgery and Neurorehabilitation, Tianjin Medical University, Tianjin; c Department of Operating Room, Xiangyang Central Hospital, Affiliated Hospital of Hubei University of Arts and Science, Xiangyang; d Department of Orthopaedic Surgery, Yangxin People’s Hospital, Yangxin, Hubei; e Department of Orthopaedic Surgery, Beijing Jishuitan Hospital, Capital Medical University, Beijing, People’s Republic of China; f Center for Musculoskeletal Surgery (CMSC), Charité-Universitätsmedizin Berlin, Corporate Member of Freie Universität Berlin, Humboldt University of Berlin, Berlin Institute of Health, Berlin, Germany

*Dear Editor,*

We read with great interest the article by Deng *et al*.^1^ published in the *International Journal of Surgery*. This study has compared the performances of three major large language models (LLMs) including GPT-4.0, GPT-3.5, and Claude2, in the clinical context of breast cancer. Their findings suggested that within the domain of clinical utilization concerning breast carcinoma, GPT-4.0 not only exhibits superiority concerning quality and pertinence but also manifests remarkable efficacy in practical implementation, notably in contrast to its predecessor, GPT-3.5. With the utilization of ChatGPT escalates, it is foreseeable that healthcare professionals and potentially individuals directly affected by medical conditions or their caregivers, will resort to this application for insights on disease prognostication, particularly in challenging prognostic scenarios^2,3^. Thus, the importance of this study deserves to be recognized.

While this study comprehensively analyzed the performance of three LLMs across five crucial domains pertaining to breast cancer, including assessment and diagnosis, treatment decisions, postoperative care, psychosocial support, as well as prognosis and rehabilitation, it is regrettable that it solely conducted descriptive examinations of LLMs performance without establishing comparative control groups comprising of professional medical practitioners. In previous study, Liu et al. investigated whether ChatGPT could respond in accordance with evidence-based medicine in neurosurgery^4^. This study encompassed 50 neurosurgical questions and posed to both GPT-3.5 and GPT-4, respectively. In addition, three neurosurgeons with varying levels of experience (low seniority, middle seniority, and high seniority), according to the guidelines set by the National Health Commission of the People’s Republic of China, were also recruited to provide answers. Analysis of results revealed that GPT-3.5 performed comparably to low seniority neurosurgeons, whereas GPT-4.0’s performance paralleled that of neurosurgeons with high seniority. In the present study, the authors chose GPT-3.5 and GPT-4.0 for testing. Therefore, we recommend that the authors at least select breast surgeons or general surgeons with low or high seniority for comparison.

In addition to this study, although many previous studies have compared the performance of ChatGPT with other LLMs in various clinical scenarios, they have also neglected to compare the performance of LLMs with humans^2,3,5^. Just as clinical diagnostic test research is often used to explore some simpler, easier, and less painful test methods to assist in disease diagnosis, ‘gold standards’ are usually used as the reference standard to evaluate the diagnostic performance of tests. We believe that the performance of human doctors could be regarded as the ‘gold standard’ and should not be ignored in most clinical testing studies on LLMs.

Finally, to the best of our knowledge, an increasing number of studies are delving into the applications of LLMs like ChatGPT in various surgical domains. We conducted preliminary searches using the keywords ‘ChatGPT’ and ‘surgery’ in the PubMed database, revealing over 680 published studies indicating a growing interest in ChatGPT within the surgical realm. However, current research methodologies are diverse and lack uniform standards. Some studies may focus on the accuracy of ChatGPT, assessing its consistency with reality through quantitative analysis and comparisons with actual data. Others may prioritize comprehensiveness, examining ChatGPT’s coverage and depth across various medical domains. Additionally, there are studies addressing readability, evaluating whether the generated texts by ChatGPT are easily understandable and acceptable. Nonetheless, the absence of a standardized evaluation framework often hampers the comparability and synthesis of these research findings, limiting a comprehensive understanding of ChatGPT’s applications in the medical field. Standardized evaluation guidelines would facilitate the improvement of the quality and safety of ChatGPT’s applications in healthcare. They could provide developers and users with clear references to better understand the strengths and limitations of ChatGPT, thus enabling a more cautious integration into clinical practice.

## Ethical approval

This study does not include any individual-level data and thus does not require any ethical approval.

## Sources of funding

This study is supported by China Postdoctoral Science Foundation (2022M720385) and Beijing JST Research Funding (YGQ-202313).

## Author contribution

H.W.: conceptualization, methodology, data curation, formal analysis, resources, investigation, and writing – original draft; W.L.: conceptualization, methodology, data curation, formal analysis, resources, and investigation; X.C.: conceptualization, methodology, data curation, formal analysis, resources, and investigation; C.L.: methodology, data curation, formal analysis, resources, investigation, and writing – review and editing.

## Conflicts of interest disclosure

The authors declare no conflicts of interest.

## Research registration unique identifying number (UIN)

1. Name of the registry: not applicable.
2. Unique identifying number or registration ID: not applicable.
3. Hyperlink to your specific registration (must be publicly accessible and will be checked): not applicable.

## Guarantor

Haiyang Wu and Cheng Li.

## Data availability statement

The data underlying this article will be shared by the corresponding author on reasonable request.
